# Simple decision-tree tool to facilitate author identification of reporting guidelines during submission: a before–after study

**DOI:** 10.1186/s41073-017-0044-9

**Published:** 2017-12-18

**Authors:** Daniel R. Shanahan, Ines Lopes de Sousa, Diana M. Marshall

**Affiliations:** 1 Faculty of 1000 Ltd., Middlesex House, 34–42 Cleveland Street, London, W1T 4LB UK; 2 BioMed Central Ltd., 236 Gray’s Inn Road, London, WC1X 8HB UK; 3 Taylor & Francis Group, 2&4 Park Square, Milton Park, Abingdon, OX14 4RN UK

**Keywords:** Reporting guidelines, Selective reporting, Pre-submission interventions, Equator

## Abstract

**Background:**

There is evidence that direct journal endorsement of reporting guidelines can lead to important improvements in the quality and reliability of the published research. However, over the last 20 years, there has been a proliferation of reporting guidelines for different study designs, making it impractical for a journal to explicitly endorse them all. The objective of this study was to investigate whether a decision tree tool made available during the submission process facilitates author identification of the relevant reporting guideline.

**Methods:**

This was a prospective 14-week before–after study across four speciality medical research journals. During the submission process, authors were prompted to follow the relevant reporting guideline from the EQUATOR Network and asked to confirm that they followed the guideline (‘before’). After 7 weeks, this prompt was updated to include a direct link to the decision-tree tool and an additional prompt for those authors who stated that ‘no guidelines were applicable’ (‘after’). For each article submitted, the authors’ response, what guideline they followed (if any) and what reporting guideline they should have followed (including none relevant) were recorded.

**Results:**

Overall, 590 manuscripts were included in this analysis—300 in the before cohort and 290 in the after. There were relevant reporting guidelines for 75% of manuscripts in each group; STROBE was the most commonly applicable reporting guideline, relevant for 35% (*n* = 106) and 37% (*n* = 106) of manuscripts, respectively. Use of the tool was associated with an 8.4% improvement in the number of authors correctly identifying the relevant reporting guideline for their study (*p* < 0.0001), a 14% reduction in the number of authors incorrectly stating that there were no relevant reporting guidelines (*p* < 0.0001), and a 1.7% reduction in authors choosing a guideline (*p* = 0.10). However, the ‘after’ cohort also saw a significant increase in the number of authors stating that there were relevant reporting guidelines for their study, but not specifying which (34 vs 29%; *p* = 0.04).

**Conclusion:**

This study suggests that use of a decision-tree tool during submission of a manuscript is associated with improved author identification of the relevant reporting guidelines for their study type; however, the majority of authors still failed to correctly identify the relevant guidelines.

**Electronic supplementary material:**

The online version of this article (10.1186/s41073-017-0044-9) contains supplementary material, which is available to authorized users.

## Background

Communicating what has been done and observed is a key aspect of the scientific process; however, there is a huge amount of evidence that much of published biomedical research is poorly reported [1].

In the early 1980s, DerSimonian and colleagues suggested a solution to this, stating that ‘editors could greatly improve the reporting of clinical trials by providing authors with a list of items that they expected to be strictly reported’ [2]. This eventually led to the development of the CONSORT Statement—a common set of recommendations for the essential items that should be included in any report of a randomised controlled trial [3, 4].

Since its publication, the CONSORT Statement has been widely shared and supported, reflected both by the number of citations received and its endorsement by major editorial organizations, for example, the International Committee of Medical Journal Editors, Committee on Publication Ethics and World Association of Medical Editors.

Despite the visibility of the CONSORT Statement, recent reviews have demonstrated that reporting of essential information continues to be generally inadequate in trial reports across all areas of medicine [5–9]. Research has also suggested that peer review—the mechanism traditionally used to ensure the integrity of the scientific literature—fails to detect important deficiencies in reporting of the methods and results of randomised trials [10].

However, there is evidence that direct journal endorsement of the CONSORT Statement can lead to important improvements in the quality and reliability of published research [11]. In their study, Turner et al. defined endorsement as a statement that implies that the CONSORT Statement is incorporated into the editorial process for the journal.

Since publication of the CONSORT Statement, there has been a proliferation of reporting guidelines for other types of research, including observational studies [12], systematic reviews and meta-analyses [13] and even case reports [14]. There are now over 350 research reporting statements available from the EQUATOR network (http://www.equator-network.org/).

A journal explicitly endorsing all these guidelines in a statement is impractical, and would make it difficult for authors to find the relevant guideline for their study, further weakening endorsement as an intervention to improve research reporting. Therefore, there is a need to identify and specifically endorse the reporting guideline relevant for an individual author’s study.

The hypothesis of this study was that a simple decision-tree tool (Penelope EQUATOR Wizard), which gathers binary information about a study from the author(s) during the submission process to calculate the study type and link them to the relevant reporting guideline for their study, would improve author identification of the relevant reporting guideline without the need to explicitly endorse it in the journal’s ‘Instructions for Authors’.

## Methods

This was a prospective before–after study to investigate the impact of a decision-tree tool to support authors in identifying the relevant reporting guidelines for their study.

### Study cohort

The study took place across four speciality medical research journals—*BMC Family Practice*, *BMC Gastroenterology*, *BMC Musculoskeletal Disorders* and *BMC Nephrology*.

A question was introduced into the submission system for each of the journals on 15 February 2016 (Table 1a), linking to the EQUATOR Network website and prompting authors to follow the relevant reporting guidelines for their study type and asking them to confirm that they have done so, or that there are no relevant guidelines for their study type. A similar statement was also added to the submission guidelines for research articles for each journal involved.

**Table 1:** Submission questions used to prompt for reporting guidelines

a) Initial question introduced to the Editorial Manager submission system Standards of reporting *[journal name]* advocates the complete and transparent reporting of research and methods. Authors are required to append the appropriate reporting guideline checklist to their manuscript on submission where applicable. Checklists are available for a number of study designs from the EQUATOR Network. See BioMed Central’s policy page for further information. Please confirm you have included as additional files the required reporting guideline checklist (and relevant extensions) and flow diagram for your study type from the EQUATOR Network. • I have followed the relevant reporting for my study type, and included a populated checklist with my submission. • Not applicable for my study.
b) Altered question after introduction of decision-tree tool Standards of reporting *[journal name]* advocates the complete and transparent reporting of research and methods. Authors are required to append the appropriate reporting guideline checklist to their manuscript on submission where applicable. Find the right guidelines for my study. Please confirm you have included as additional files the required reporting guideline checklist (and relevant extensions) and flow diagram for your study type from the EQUATOR Network. See BioMed Central’s policy page for further information. • I have followed the relevant reporting for my study type, and included a populated checklist with my submission. • Not applicable for my study. *[to c]*
c) Sub-question prompted by authors stating there were no applicable reporting guidelines The EQUATOR Network have been working with Penelope to create a tool to help authors find and use the relevant reporting guidelines for their study. Find the right guidelines for my study. • I have followed the relevant reporting for my study type, and included a populated checklist with my submission. • There are no relevant guidelines for my study type.

After 7 weeks, this question was updated to include a link to the decision tool on 4 April 2016, including a second prompt for those authors who stated that there were no relevant guidelines for their study type (Table 1b, c). This question was then removed after another 7 weeks, on 23 May 2016.

Manuscripts were defined as part of the ‘before’ or ‘after’ group depending on what question they answered during submission.

### Intervention

The intervention was the Penelope EQUATOR Wizard (http://www.peneloperesearch.com/equatorwizard/). This automated decision tree asks authors to provide yes–no answers regarding their study to calculate the study type and the relevant reporting guideline. The decision tree for the tool can be seen in Additional file 1; it includes 11 commonly-used guidelines:

- Animal Research: Reporting of In Vivo Experiments (ARRIVE) [15]
- CAse REport (CARE) guidelines [14]
- Consolidated Standards of Reporting Trials (CONSORT) [4]
- ENhancing Transparency in REporting the synthesis of Qualitative research (ENTREQ) [16]
- Meta-analysis Of Observational Studies in Epidemiology (MOOSE) [17]
- Preferred Reporting Items for Systematic Reviews and Meta-Analyses (PRISMA) [13]
- REporting recommendations for tumour MARKer prognostic studies (REMARK) [18]
- Standards for Reporting Qualitative Research (SRQR) [19]
- Standards for Reporting of Diagnostic Accuracy (STARD) [20]
- Strengthening the Reporting of Observational Studies in Epidemiology (STROBE) [12]
- Transparent Reporting of a multivariable prediction model for Individual Prognosis or Diagnosis (TRIPOD) [21].

### Outcomes

The primary outcome for this study was the percentage of authors who identified the correct reporting guidelines for their study type.

For each article submitted, we recorded the authors’ response to the submission question, what guideline they followed (if any) and what reporting guideline they should have followed (including none relevant). Study protocols and economic evaluations, which are listed by the EQUATOR Network as ‘main study types’ but whose reporting guidelines were not included in the decision tree were excluded from the analysis. Those studies that had explicitly and appropriately followed reporting guidelines that were not included in the decision tree were also excluded.

The reporting guidelines followed by the authors were identified from the full text of the manuscript—this included direct references or mentions of the guidelines followed, submission of a reporting checklist or inclusion of the relevant flow diagram. Author adherence to the reporting guidelines and completeness of reporting were not evaluated as a part of this study.

Each submitted manuscript was independently evaluated by two of the investigators (DRS, DM or ILS) to identify the study type and what reporting guideline should have been followed. In the event of a disagreement between the two investigators, a consensus was reached between the group.

Each manuscript was classified as one of six possible outcomes:

- Authors identified the correct reporting guideline;
- Authors correctly stated that no reporting guidelines were relevant to their study type;
- Authors correctly identified that there were reporting guidelines relevant to their study type, but provided no information as to which;
- Authors followed a reporting guideline that was inappropriate for their study type;
- Authors incorrectly stated that no reporting guidelines were relevant for their study type;
- Authors incorrectly stated that there were relevant reporting guidelines for their study type.

### Statistical analysis

Data were analysed using Microsoft Excel 2010. Analyses were conducted for each outcome separately; the percentage of manuscripts in each possible outcome was recorded both before and after the introduction of the decision-tree tool. One-tailed Student’s *t* test for proportions was used to evaluate the difference between the proportions, with *α* < 0.05 and *H*_0_ that there were no differences between the proportions.

## Results

Overall, 611 manuscripts were submitted during the study period, with 590 included in the analysis—300 in the before cohort and 290 in the after; 10 and 11 manuscripts were excluded from each cohort, respectively as they concerned study types that had well-established reporting guidelines that were not included in the decision-tree tool. There were no significant differences between the two cohorts at baseline (Table 2).

**Table 2:** Baseline characteristics of each cohort

	Before	After	∆
No. of manuscripts submitted in timeframe	310	301	− 9
No. of manuscripts included in analysis	300	290	− 10
No. with applicable reporting guidelines	224 (75%)	217 (75%)	0.2%
ARRIVE	18 (6.0%)	14 (4.8%)	− 1.2%
CARE	38 (13%)	24 (8.3%)	− 4.4%
CONSORT	10 (3.3%)	20 (6.9%)	3.6%
ENTREQ	2 (0.67%)	0 (0.0%)	− 0.7%
MOOSE	3 (1.0%)	0 (0.0%)	− 1.0%
PRISMA	18 (6.0%)	21 (7.2%)	1.2%
REMARK	11 (3.7%)	4 (1.4%)	− 2.3%
SQUIRE	0 (0.0%)	2 (0.07%)	0.7%
STARD	6 (2.0%)	12 (4.1%)	2.1%
SRQR	12 (4.0%)	12 (4.1%)	0.1%
STROBE	106 (35%)	106 (37%)	1.2%
TRIPOD	0 (0.0%)	2 (0.07%)	0.7%

There were relevant reporting guidelines for 75% of manuscripts in each group (*n* = 224 in the before cohort, *n* = 217 in the after cohort). The most commonly applicable reporting guideline was STROBE, which was relevant for 35% (*n* = 106) of manuscripts submitted in the before cohort, and 37% (*n* = 106) of manuscripts in the after cohort (Table 2).

Overall, use of the tool was associated with a statistically significant 8.4% improvement in the number of authors correctly identifying the relevant reporting guideline for their study (Table 3; *p* < 0.0001). Similarly, there was an improvement in the number of authors incorrectly stating that there were no relevant reporting guidelines for their study (37% before vs 23% after; *p* < 0.0001) and a reduction in the number of authors choosing a reporting guideline that was not applicable to their study on submission; however, this was not statistically significant (3.1 vs 1.4%; *p* = 0.10).

**Table 3:** Summary outcomes from the two cohorts

Outcome	Before	After	∆	*p* value
No. of manuscripts included in analysis	300	290	− 11	–
No. with applicable reporting guidelines	224 (75%)	217 (75%)	0.2%	0.96
Correct reporting guideline identified	70 (31%)	86 (40%)	8.4%	<0.0001
Identified the incorrect reporting guideline	7 (3.1%)	3 (1.4%)	− 1.7%	0.10
Incorrectly stated that none are relevant	82 (37%)	49 (23%)	− 14%	<0.0001
Correctly stated that relevant reporting guidelines exist, but not which	65 (29%)	79 (36%)	7.4%	0.04
No. without applicable reporting guidelines	76 (25%)	73 (25%)	− 0.2%	0.96
Correctly stated none relevant	50 (66%)	47 (64%)	− 1.4%	0.65
Incorrectly stated that relevant reporting guidelines exist, but not which	26 (34%)	26 (36%)	1.4%	0.48

Overall, the number of authors who correctly stated that there were no relevant reporting guidelines for their study was comparable between the two groups (66 vs 64%; *p* = 0.65), as were the proportion of authors incorrectly stating that there were relevant reporting guidelines for their study, but not indicating which (34 vs 36%; *p* = 0.48).

Combining those authors who correctly identified the relevant reporting guidelines and those who correctly stated that there were no relevant reporting guidelines for their study type shows an increase of 6% in the after cohort (40 vs 46%).

## Discussion

A large systematic review involving 50 studies and reports of more than 16,000 randomised trials demonstrated that journal endorsement of the CONSORT checklist was associated with an improvement in the completeness of reporting for 22 of 25 CONSORT checklist items [11]. However, endorsement as an intervention is poorly defined. A recent review by Shamseer et al. on high-impact-factor medical journals demonstrated that 63% (106/168) of the included journals mentioned CONSORT in their ‘Instructions to Authors’, 42% (*n* = 44) explicitly stated that authors ‘must’ use CONSORT to prepare their trial manuscript, and 38% required an accompanying completed CONSORT checklist as a condition of submission [22].

Inadequate reporting is also a huge problem in study types other than randomised trials, including systematic reviews [23], diagnostic studies [24], animal studies [25], observational studies [26], clinical predication studies [27], qualitative studies [28] and surveys [29, 30], contributing to an estimated $85billion wasted annually [31].

To our knowledge, no other studies have been conducted to evaluate the impact of endorsement as an intervention to improve the reporting of published research. This study suggests that use of a simple decision-tree tool during manuscript submission facilitated author identification of the relevant reporting guidelines for their study type. However, even with use of the tool, the majority of authors failed to identify the correct reporting guideline for their study.

One possible explanation for this could be that prompting authors regarding reporting requirements at the point of submission is too late in the publication process, as they will have already written their manuscript. This is supported by the observed increase in the number of authors stating that they had followed the relevant reporting guideline, without presenting any evidence to support this, following the introduction of the tool, and the significant decrease in the number of authors incorrectly stating that there were no relevant reporting guidelines for their study.

This could suggest that the change in the question asked influenced authors’ behavior during article submission, with their ‘default’ answer changing depending on the formulation of the question, rather than the tool influencing how they reported their study. Furthermore, due to the submission system used (Editorial Manager), it was not possible to track which authors actually used the tool during the submission system, which prevents us from strongly correlating use of the tool with an improvement in author identification of reporting guidelines.

As this analysis only concerned the identification of the relevant reporting guideline, not the completeness of reporting of the manuscript, it is not possible to evaluate the impact of the tool on the completeness of the literature. However, the association demonstrated between endorsement of the CONSORT Statement and the completeness of published clinical trials suggests that it could have such an improvement [11], although further research would be needed to confirm this effect and whether it was meaningful.

## Conclusion

This before–after study suggests that use of a decision-tree tool during submission of a manuscript is associated with improved author identification of the relevant reporting guidelines for their study type; however, the majority of authors still failed to correctly identify the relevant guidelines.

## Additional file

Additional file 1: EQUATOR reporting guideline decision tree. (PDF 234 kb)
