# Supplementary figures and images for: Simple decision-tree tool to facilitate author identification of reporting guidelines during submission: a before–after study
Source: Res Integr Peer Rev. 2017 Dec 18;2:20. doi: 10.1186/s41073-017-0044-9 (PMC5803581; doi:10.1186/s41073-017-0044-9)

# EQUATOR Reporting Guideline Decision Tree

## Which guidelines are relevant to my work?

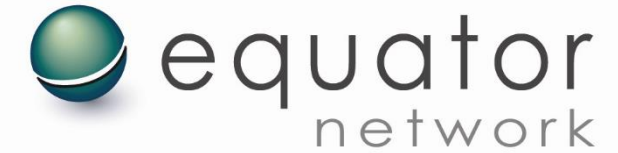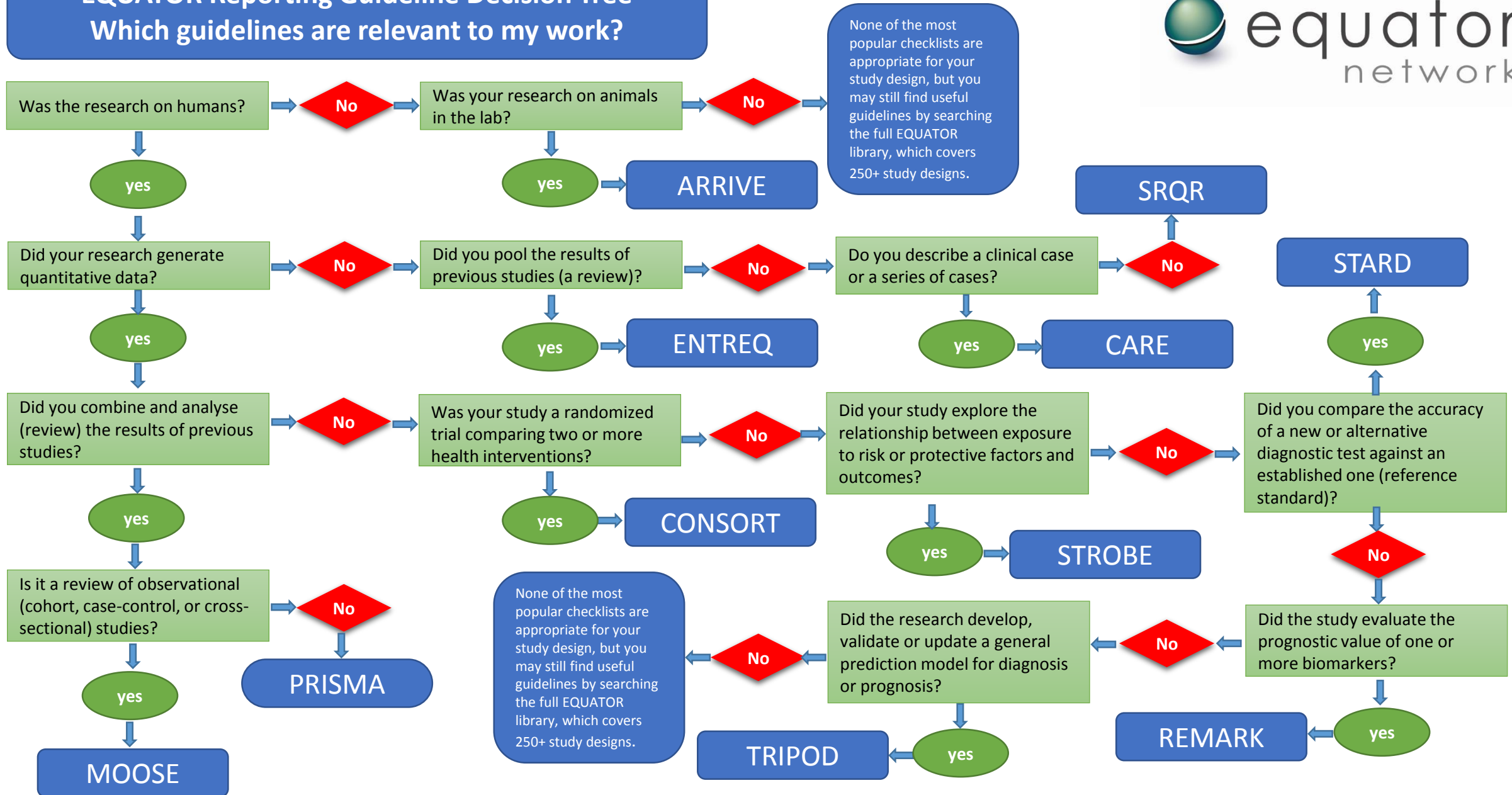

Supplement: Additional file 1: — EQUATOR reporting guideline decision tree. (PDF 234 kb) [file 41073_2017_44_MOESM1_ESM.pdf]
